# Membrane protein assembly: two cytoplasmic phosphorylated serine sites of Vpu from HIV-1 affect oligomerization

**DOI:** 10.1038/srep28866

**Published:** 2016-06-29

**Authors:** Chin-Pei Chen, Meng-Han Lin, Ya-Ting Chan, Li-Chyong Chen, Che Ma, Wolfgang B. Fischer

**Affiliations:** 1 Institute of Biophotonics, School of Biomedical Science and Engineering and Biophotonics & Molecular Imaging Center (BMIRC), National Yang-Ming University, Taipei 112, Taiwan; 2 Genomics Research Center, Academia Sinica, Taipei 115, Taiwan; 3 Center for Condensed Matter Sciences, National Taiwan University, Taipei 106, Taiwan

## Abstract

Viral protein U (Vpu) encoded by human immunodeficiency virus type 1 (HIV-1) is a short integral membrane protein which is known to self-assemble within the lipid membrane and associate with host factors during the HIV-1 infectivity cycle. In this study, full-length Vpu (M group) from clone NL4-3 was over-expressed in human cells and purified in an oligomeric state. Various single and double mutations were constructed on its phosphorylation sites to mimic different degrees of phosphorylation. Size exclusion chromatography of wild-type Vpu and mutants indicated that the smallest assembly unit of Vpu was a dimer and over time Vpu formed higher oligomers. The rate of oligomerization increased when (i) the degree of phosphorylation at serines 52 and 56 was decreased and (ii) when the ionic strength was increased indicating that the cytoplasmic domain of Vpu affects oligomerization. Coarse-grained molecular dynamic simulations with models of wild-type and mutant Vpu in a hydrated lipid bilayer supported the experimental data in demonstrating that, in addition to a previously known role in downregulation of host factors, the phosphorylation sites of Vpu also modulate oligomerization.

The generation of functional forms of membrane proteins comprises several steps: membrane insertion during the translation process *via* the translocon complex^1^ or other systems^2^, and the proper assembly of the proteins into a quaternary structure, if necessary. It has been asserted that after insertion into the membrane, proteins undergo structural arrangements in the monomeric form. In an analogy with single protein folding, during synthesis proteins are thought to rapidly achieve an intermediate state referred to as the ‘molten globule’ or ‘compact intermediate’ state^3^. Since hardly any information is available about this state, at this point, how the final assembly is formed can only be speculated.

Viral channel forming proteins (VCPs) encoded by the virus are a special type of membrane protein which are a dependant of the larger ion channels of the host^4^,^5^,^6^,^7^,^8^ but smaller in size. Since VCPs are also known to interact with host proteins and initiate ion channel-independent functions, it can be hypothesized that they also need to ‘exist’ as monomers. In this respect, VCPs can be used to explore the dynamics and structural features of membrane protein assembly within the lipid membrane^9^,^10^,^11^,^12^.

Vpu of HIV-1 is one of VCPs with 81 amino acids in length and contains a single helical transmembrane domain (TMD)^6^,^13^ followed by a cytoplasmic domain consisting of another two helices and further residues towards the C terminal side^13^,^14^,^15^,^16^. The ion channel activity of Vpu has been shown to be attributed solely to the TMD^17^. A recent review has discussed speculations about the, as yet, unclear ion channel function of Vpu *in vivo*^18^. In addition, Vpu is phosphorylated at two serines at positions 52 and 56 which are responsible for initiating downregulation of membrane proteins of the host, including CD4^19^, BST-2^20^,^21^ or NTB-A^22^. This function of initiation of host factor degradation is independent of the function of altering electrochemical gradients *via* the formation of an ion channel^17^,^23^.

The oligomeric state of Vpu has not been univocally established. While gel permeation chromatography suggests that a maximum of five proteins are assembled^24^. Computational models which were based on NMR spectroscopic data show structural features of a tetrameric or pentameric form of the TMD of Vpu^10^.

At present, the known architecture of ion channels based on crystallographic data suggests that hydrophilic residues face the lumen of a putative ion conducting pore (see for example^25^). In the case of the pentameric ligand gated ion channel of *Gloebacter violaceus* (GLIC), the serines and threonines of the pore-lining helices M2 of each of the five subunits points into the lumen forming a hydrophilic ring^25^. It was also speculated that the only hydrophilic residue in the transmembrane domain of Vpu, Ser-23, should point into an ion conducting pore^26^. However, in these computational models^10^ Ser-23 is located at the helix-lipid interface leaving the putative pore as a pure hydrophobic stretch, they contradict the current notion of the putative pore architecture. Consequently, there is a need for further refinement of the model of the formation of ion-conducting pore by assembled Vpu. In addition, Vpu is known to act against host factors for down-regulation. Vpu was proposed to exist in a stable equilibrium between oligomeric and monomeric forms, which are inactive and active, respectively, for interacting with host proteins^27^. However, how Vpu is assembled and how it eventually reaches a pore-like formation remains to be characterized.

In this study, we investigated the oligomeric behavior of Vpu expressed in human HEK 293 cells and purified into detergents micelles to retain its tertiary folding. Wild-type (WT) Vpu and mutations at the sites of the phosphorylated serines at positions 52 and 56 were investigated to assess the role of phosphorylation in the dynamics of assembly. Coarse grained molecular dynamics (CGMD) simulations of Vpu proteins embedded in a planar lipid bilayer model were chosen to evaluate the oligomeric assembly under likely *in vivo* conditions such as an abundance of Vpu proteins in a large lipid patch and simulated over a long time period. In addition, CGMD simulations proposed mechanical features of how individual domains of Vpu, both transmembrane and cytoplasmic, contribute to the assembly process.

## Results

### Protein dimers and higher oligomers in detergent micelles

**Figure 1:**
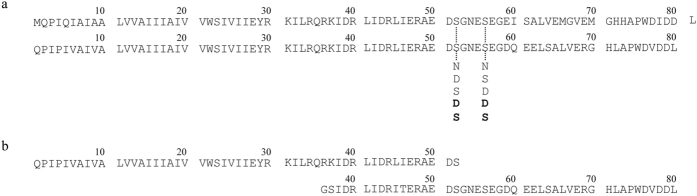
Amino acid sequences of Vpu. Amino acid sequences of Vpu-WT and its mutants used in experiments (upper line) and the computational models (lower line) (**a**). Sequences used in the computational model Vpu_1–52_ (upper line) and used in the NMR experiment of PDB ID 2K7Y (lower line) (**b**). The mutations at sites Ser-52/56 are shown with the mutations used in the computational model shown in bold.

**Figure 2:**
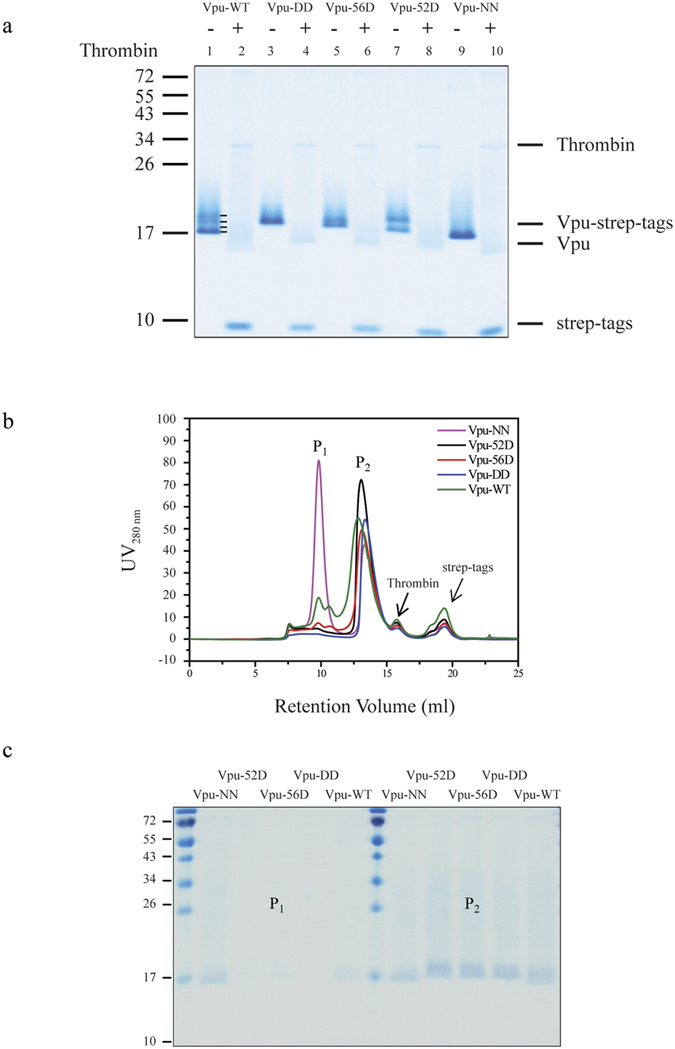
Two oligomeric states of wild-type and different mutants of full-length Vpu (pNL4.3) from HIV-1. Coomassie stained SDS-PAGE analysis of purified Vpu-WT, Vpu-DD, Vpu-56D, Vpu-52D, Vpu-NN protein elution from left to right (**a**). All fusion proteins were treated with thrombin enzyme to remove the fusion strep-tags. The “+” and “−” represent Vpu fusion proteins with or without the addition of thrombin enzymes. Molecular mass is given in kDa. Size exclusion chromatograms of Vpu-WT (green curve), Vpu-DD (blue curve), Vpu-56D (red curve), Vpu-52D (black curve), Vpu-NN (pink curve) (**b**). SDS-PAGE analysis of the highest intensity fractions of big oligomers (P_1_) and small oligomers (P_2_) from size exclusion chromatographs of wild-type and different mutants of Vpu proteins (**c**).

Vpu-WT and mutant Vpu proteins were expressed in HEK 293 cells (Fig. 1). SDS-Page analysis from cells expressing Vpu-WT revealed four bands (Fig. 2a, lane 1). The SDS-PAGE analysis of the double mutants Vpu-DD and Vpu-NN, which lack phosphate groups at the serines, showed only a single band each on the SDS-PAGE at various molecular weights due to the decreased migration rate of the negative charged Vpu-DD upon denaturation (Fig. 2a, lanes 3 and 9)^28^,^29^. Vpu-52D and Vpu-56D each show two bands (Fig. 2a lanes 5 and 7). Taken together, these results indicate that the four bands of Vpu-WT represent the following from high to low molecular weight, (i) phosphorylation of both of the serines, Ser-52 and Ser-56, (ii) single phosphorylated serines at position 56 and (iii) position 52, and (iv) fully non-phosphorylated serines. The ratio between phosphorylated and non-phosphorylated Vpu remains the same as found in measurements directly from cell pellets using anti-strep-tag antibody for Vpu in Western blot (data not shown).

The thrombin enzyme cleaves the strep-His8 fusion tag from Vpu-WT and the mutants (Fig. 2a lanes 2, 4, 8 and 10 and Supplementary Fig. S1). The pattern mentioned for uncleaved Vpu does not seem to be affected by thrombin treatment.

The fusion tag-free Vpu was further purified by size-exclusion chromatography and eluted with four peaks (Fig. 2b). Vpu-WT showed two peaks, a smaller peak at 9.5 ml representing large protein/detergent complexes (P_1_ in Fig. 2b) and a larger peak representing smaller protein/detergent complexes (P_2_ in Fig. 2b) at 13.5 ml. Mutant Vpu-56D shows a similar pattern. For Vpu-52D and Vpu-DD the peak of the large complexes was not resolved. Vpu-NN showed the peak of the large complexes being larger than that of the smaller complexes. SDS-PAGE analysis identified the two peaks representing Vpu protein and its respective mutations (Fig. 2c). The third and fourth peaks correspond to thrombin and strep-His8 fusion tags, respectively.

Multi-angle light scattering analysis identified that P_1_ and P_2_ correspond to molecular weights of 174.7 ± 18.4 kDa and 18.0 ± 1.9 kDa, respectively (Table 1). The respective averaged oligomeric state was calculated to be around 19.0 ± 2.0 for P_1_ and 2.0 ± 0.2 for P_2_. Thus, Vpu is able to exist in two oligomeric states, which are most likely a dimer and higher oligomer.

### Modulation of the dynamics of Vpu-WT, Vpu-NN and Vpu-DD oligomerization by the two phosphorylation sites

**Figure 3:**
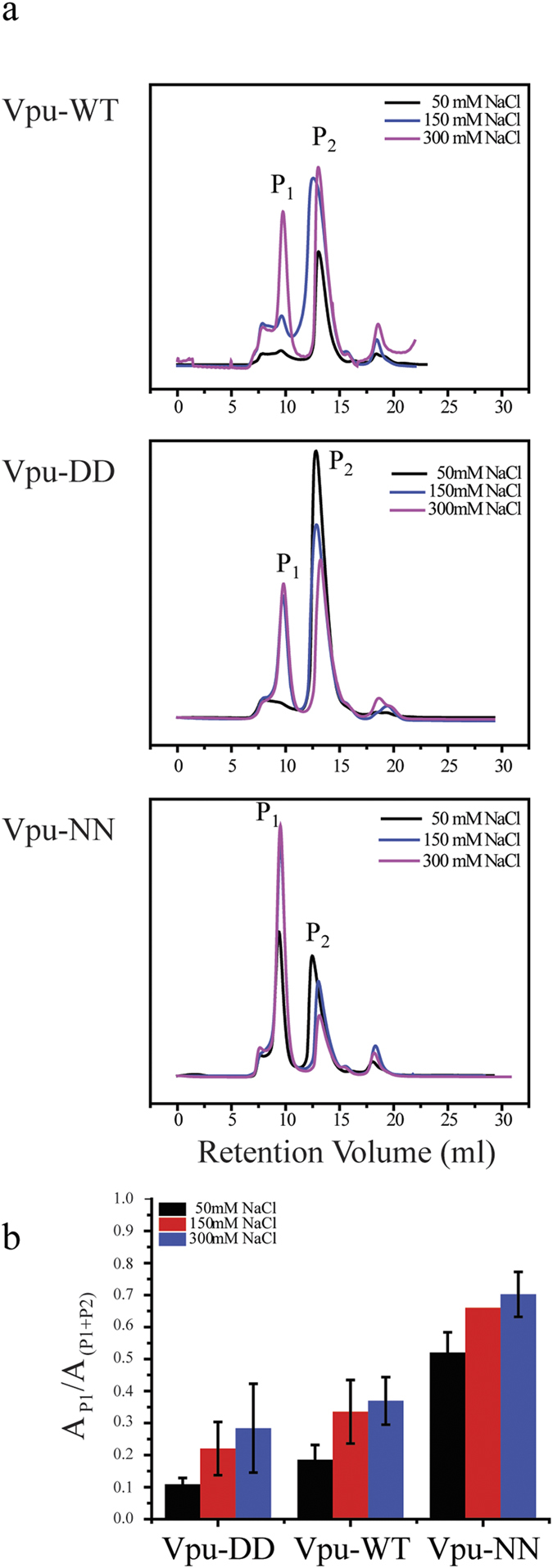
Dynamics of Vpu-WT, Vpu-NN, and Vpu-DD oligomerization in different salt solution using size exclusion chromatograms (SEC). (**a**) SEC profiles of Vpu-WT, Vpu-NN, and Vpu-DD in different salt buffer concentration (50 mM NaCl, 150 mM NaCl and 300 mM NaCl). (**b**) Comparison of peak area, P_1_/(P_1_ + P_2_), with different salt concentrations of Vpu-WT, Vpu-NN, and Vpu-DD.

After purification of the proteins from a stock solution, the peak ratio between the higher oligomer (referring to P_1_) and the dimer (referring to P_2_) for Vpu-WT and Vpu-DD was in favor of the dimer for all ionic strengths investigated, 50, 150 and 300 mM NaCl (Fig. 3a). The peak of the higher oligomer is the largest at the highest ionic strength of 300 mM NaCl for the two proteins. The peak area of the higher oligomer was largest for Vpu-NN at all ionic strengths (Fig. 3b). Higher ionic strength screens the negative charges at the serine sites and even the partial charges of the amide group in asparagine indicating electrostatic type interaction modulates assembly.

**Figure 4:**
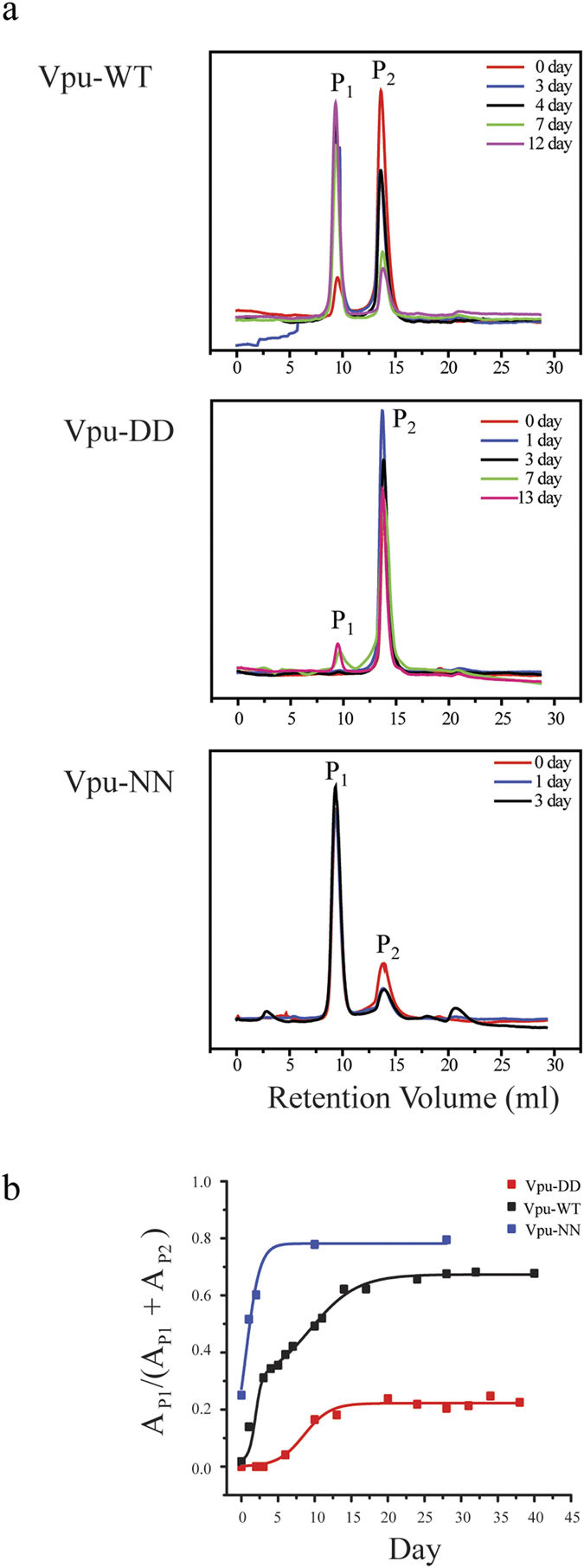
Dynamics of two oligomeric states of Vpu-WT, Vpu-NN, and Vpu-DD using size exclusion chromatograms. (**a**) Time-dependent SEC profiles of Vpu-WT, Vpu-NN, and Vpu-DD in 50 mM NaCl buffer concentration. (**b**) The variation of day peak area, P_1_/(P_1_ + P_2_), of Vpu-WT, Vpu-NN, and Vpu-DD. Data are fitted according to (1) as outlined in Materials and Methods.

Immediately after purification of Vpu-WT from a stock solution, the peak of the dimer was larger than that of the higher oligomer (Fig. 4a, top graph). Repeating the purification from the stock solution over a period of 12 days revealed a gradual increase in the higher oligomer. A slower increase in the peak of the higher oligomer was observed for Vpu-DD. Purification after 7 days showed just the beginning of a small peak for the dimer (Fig. 4a, middle graph, green line). In the case of Vpu-NN, the peak of the higher oligomer was larger than that of the dimer from the first day of the experiment and increased even more over three days, finally reaching a plateau over a longer period (Fig. 4a, lower graph).

The dynamics data was plotted as area of the peak of the higher oligomer (A_P1_), divided by the total area of A_P1_ and the peak area of the dimer (A_P2_), A_P1_/(A_P1_ + A_P2_), over time for Vpu-WT with a double logarithmic growth curve (Fig. 4b, black and Table 2). Vpu-DD (Fig. 4b, red) and Vpu-NN (Fig. 4b, blue) can both be fitted with a single function (see also Table 2). Vpu-NN (c = 1.05 day^−1^) and Vpu-DD (c = 0.56 day^−1^) mark a fast and slow increase in the area of the higher oligomer, respectively. Vpu-WT exhibited a fast increase (c = 2.35 day^−1^) first, followed by a slow increase (c = 0.31 day^−1^), similar to the afore-mentioned growth rates of Vpu-NN and Vpu-DD, respectively. As a result, Vpu-DD, due to their negative charges at the two serine sites tended to assemble very slowly reaching a final assembly ratio of a = 0.22, whilst Vpu-NN, having charges removed at the site of the two serines, assembled very quickly reaching the largest assembly ratio of a = 0.78. Therefore, the fast increase in the peak of the higher oligomer of Vpu-WT should be due to the assembly of non-phosphorylated Vpu, whilst the slower increase of that peak should be due to the assembly of both, single and double phosphorylated Vpu proteins. The single phosphorylated Vpu proteins obscure the plot in as much they show ‘mixed’ assembly dynamics. The negative charges of the phosphorylated serine site slowdown or even prevent oligomerization.

### Sequence of mechanical events upon oligomerization by CGMD simulations of Vpu-WT and Vpu DD in hydrated lipid bilayers

**Figure 5:**
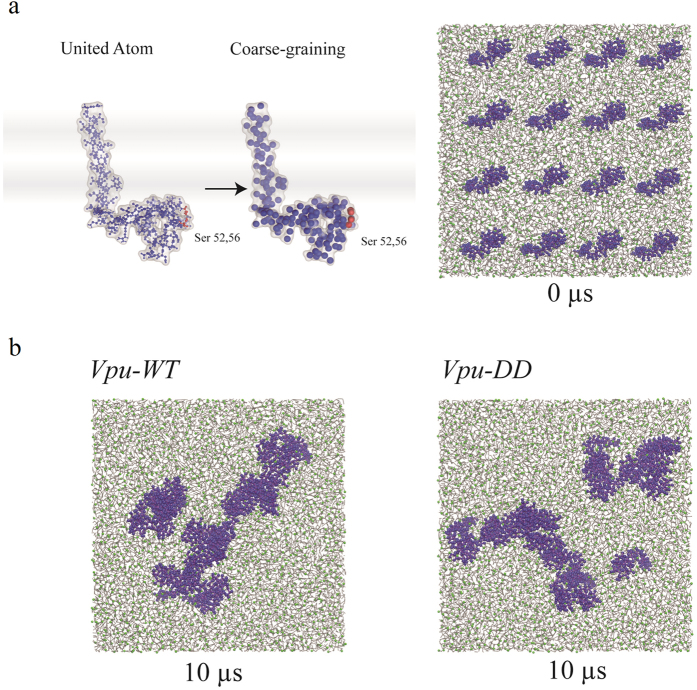
Assembly of Vpu within lipids using CGMD simulations. (**a**) The full-length Vpu, Vpu_1–82_, is built from experimental structures from NMR spectroscopy (PDB ID: 2K7Y) of the cytoplasmic domain and the formation of an ideal transmembrane helix (left). Vpu_1–80_ is transformed to a coarse-grained (CG) model structure after applying MD simulations (left). The two serines at positions 52 and 56 are shown in red. Position of Ser-23 is indicated by an arrow. In total, 16 Vpu models are inserted into a lipid membrane (right, at 0 μs). (**b**) CGMD simulations of sixteen *Vpu-WT* and *Vpu-DD* (blue) embedded in DOPC lipid patch after 10 μs.

The computational model of Vpu was generated by bending a helical motif of Vpu_1–52_ at the site of the EYR motif as reported previously^30^ (Fig. 5a, left). Sequence alignment shows that the strains used for building the computational models and the one used in the experimental study share 79% sequence identity (data not shown). Two copies of the kinked Vpu_1–52_ are run in a single lipid patch in an inverted orientation for 100 ns (Fig. S1). Both of the helices remain in both of the structures. Both of the structures (Fig. S1, black and red curves) show larger root mean square fluctuation (RMSF) values for residues Glu-28 to Ile-32. The structure shown by the red lines in Fig. S1, named Vpu_1–52_, was chosen for the next step, since the residues Leu-33 to Arg-40 of its second membrane-associated helix show lower RMSF values than those of the structure represented by the black curve. Residues Ile-38 to Ala-49 of the second, membrane-associated helix of Vpu_1–52_ are overlapped with the N terminal side of the NMR-based structure of Vpu_36–81_^16^ to finally generate full-length Vpu_1–80_ with united atoms. MD simulation of two copies of Vpu_1–80_ showed a leveling off of the root mean square deviation (RMSD) values after about 10 ns (Fig. S2, upper left). One of the Vpu_1–80_ structures showed large fluctuations of the amino acids in the kink region (Ile-32 to Gln-35, Fig. S2, upper right, red curve). These residues define the intermediate parts between the helices.

Based on these values and the leveling of the RMSD values this structure was considered further for CGMD simulations as *Vpu-WT* (Fig. 5a, left and Fig. S3). In *Vpu-WT* the serines are not phosphorylated. At this stage the CG mutant model *Vpu-DD* was generated by replacing the two serines with two aspartic acids. A total of 16 *Vpu-WT* and *Vpu-DD* are embedded in a hydrated lipid bilayer (0 ns, Fig. 5a, right) and simulated for 10 μs (Fig. 5b). The 16 *Vpu-WT* started to assemble into two large units consisting of 3 and 13 proteins (Fig. 5b, left). *Vpu-DD* at the end of the simulation shows three units of 1, 6 and 9 proteins (Fig. 5b, right).

**Figure 6:**
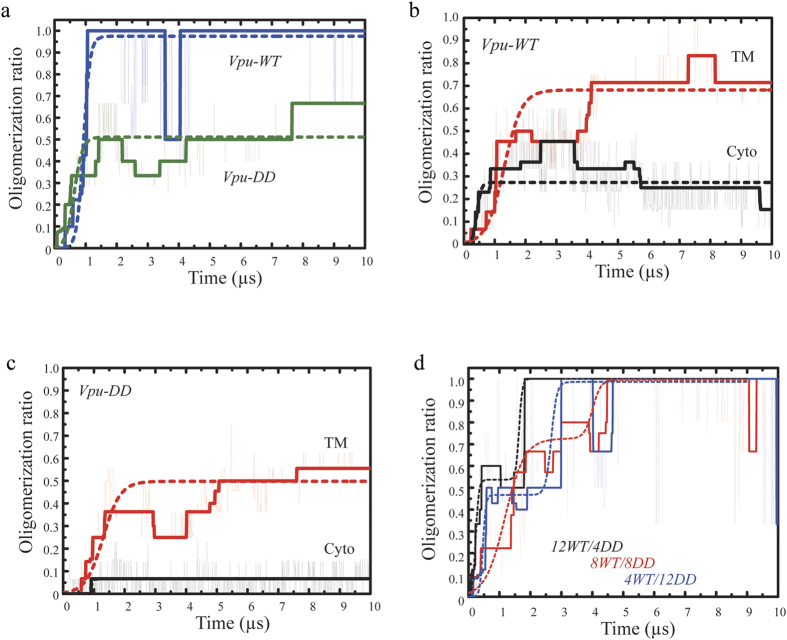
Comparison of the oligomerization ratio from CGMD simulations. (**a**) Time-dependent representations of the total oligomerization ratio of *Vpu-WT* (blue) and *Vpu-DD* (green) as well as separated into the ratio of the transmembrane domain (red curves) and the cytoplasmic domains (black curves) of *Vpu-WT* (**b**) and *Vpu-DD* (**c**). Data are fitted according to (1) as outlined in Materials and Methods for (**a–c**). Time dependent representation of the total oligomerization ratio of mixtures of *Vpu-WT* and *Vpu-DD*: 12 *Vpu-WT* and 4 *Vpu-DD* (*12WT/4DD*), 8 *Vpu-WT* and 8 *Vpu-DD* (*8WT/8DD*) as well as 4 *Vpu-WT* and 12 *Vpu-DD* (*4WT/12DD*) in the simulation box. Data are fitted with a double logarithmic curve (see Supplementary Table S2).

After about 1 μs, *Vpu-WT* reached an oligomerization ratio of nearly 1 (a = 0.98, Table 2), compared to *Vpu-DD* which reached a value of about a = 0.58 (Fig. 6a and Table 2). The oligomerization ratio of *Vpu-WT* was due to large values of both TMD assembly (a = 0.68) and the cytoplasmic domain (a = 0.27) (Fig. 6b). For *Vpu-DD* as well, the TMD assembly contributed the most (a = 0.50) to the overall oligomerization compared to the cytoplasmic domain (a < 0.1) (Fig. 6c). Analysis of the growth curve showed that the growth rates c of the TMDs are almost independent of the phosphorylation state. The higher growth rate of the cytoplasmic domains of *Vpu-DD* compared to the rate of *Vpu-WT* is due to an almost sudden assembly of a few proteins (Table 2). In this state the growth rates were not compared with those of the experiments due to the different time scales.

Long lasting dimers of both Vpu-WT and Vpu-DD form close contact areas within the TMD along the line of valines (residues 6 to 13) of one monomer with the leucines and isoleucines of the other monomer^31^. Pore like structures with eventually serines (Ser-23 of the TMD) pointing towards the center of a putative pore have not been observed.

A striking feature is that assembly of *Vpu-WT* is driven by an early assembly of the cytoplasmic domain within 0.5 μs to an oligomerization ratio of ∼0.28 followed by an increasing rate of assembly due to the TMD within the first micro second of up to ∼0.70 (Fig. 6b). For *Vpu-DD* the sequence is reversed by assembly *via* TMDs of up to ∼0.50 oligomerization followed by cytoplasmic assembly which remains a ratio of ∼0.05 (Fig. 6c).

The oligomerization ratio of mixtures of *Vpu-WT* and *Vpu-DD* (12 *Vpu-WT* and 4 *Vpu-DD*, 8 *Vpu-WT* and 8 *Vpu-DD* as well as 4 *Vpu-WT* and 12 *Vpu-DD*) achieve maximum level at a later time step as for the ‘pure’ systems (Fig. 6d). Deriving the growth rate, c, from a fitting of the curves with a double logistic growth function indicates that in all the mixtures the first rate is faster than the second rate except for the mixture of 12 *Vpu-WT* and 4 *Vpu-DD* (Supplementary Table 2). Oligomerization of the TMDs does not follow this trend due to internal reorientations within the patches (Supplementary Table 2 and Supplementary Fig. S5).

Rate of oligomerizaion is driven by the assembly of the TMD of Vpu independent of negative charges due to phosphorylation of the two serines 52 and 56, while maximum degree of oligomerization depends on the negative charges.

## Discussion

In this study, full-length Vpu from clone NL4-3 was overexpressed in HEK 293 cells using an experimental protocol in which the protein never leaves the lipid or lipid-like environment. The precise oligomeric state of Vpu has not yet been established. In many studies, synthetic peptides corresponding to the transmembrane domain of Vpu or Vpu expressed in *Escherichia coli*^10^ and purified have been used (Supplementary Table S1). In these studies using electrophoresis, it was proposed that the TM of Vpu^32^ exists in the tetramer to hexamer range. Size-exclusion chromatography of full-length Vpu by coupled transcription/translation systems suggests a pentameric structure^24^. In the *E. coli* system, all forms of Vpu, either the TM-containing segment or full-length Vpu, were over-expressed into inclusion bodies and extracted into denaturating buffer. In a subsequent step the Vpu protein is then refolded. In this study, full-length Vpu is expressed in human cells and extracted into LDAO micelles from cell membranes. LDAO is a gentle and commonly-used detergent for membrane protein structure determination^33^,^34^. Application of other detergents like Cymal5 (5-cyclohexyl-1-pentyl-ß-D-maltoside), also showed a similar pattern of two peaks in the size-exclusion chromatogram (data not shown). The expression system and purification steps in this protocol most likely keep the structure in the native states as much as was possible. In addition, SEC-MALS which measures molar mass directly is used as a tool to obtain the oligomeric states of Vpu without relying on reference standards which are usually needed in conventional size-exclusion chromatography. From our results, the smallest oligomeric state of Vpu and Vpu with phosphate group is a dimer. Experimental evidence about this has not been reported in previous studies. The dimer is assembling into larger assemblies of up to approximately 19 proteins.

Mutating Vpu-WT into Vpu-NN is chosen as a way to remove phosphorylation sites in this protein since this technique is anticipated to maintain the overall structure of the protein^35^,^36^,^37^,^38^.

### Comparison of computational and experimental data

The Vpu model in respect to its cytoplasmic domain relies on NMR spectroscopic investigations in which the peptide is non-phosphorylated^16^. The structural feature is of two helices connected by a loop, which harbors the two serine sites 52 and 56. Another study in which a much shorter peptide, Vpu_41-62_, is used indicates that a short helical part towards the C terminal side disappears upon phosphorylation but the overall shape of a loop conformation remains^39^,^40^. Thus, the CG models Vpu-WT and Vpu-DD reflect reliable structural features.

The computational system is designed to represent an estimate of the *in vivo* system. The proteins are embedded within a planar lipid bilayer of a single type of lipid molecule. Thus, the question of whether the Vpu proteins would oligomerize in the same way and with the same dynamics when embedded in a lipid bilayer can be addressed. In this study, the computational models exhibit the same behavior as found experimentally. The dimer is smallest unit to assemble. The level and growth rate of oligomerization of Vpu without the phosphate groups is bigger and faster than Vpu with phosphate groups. In addition, structural features taken from the simulation data allowed specification of the interaction dependent on the cytoplasmic domain and TMD with the latter contributing mostly to the oligomerization ratio. The coarse-graining investigations of conformational dynamics are limited to emphasizing the diffusive aspects of the protein in the bilayer. The computational data represent a semi-quantitative analysis of protein diffusivity which matches the experimental findings. The number of Vpu was chosen to be 16 instead of the putative 19 Vpu molecules calculated from the experimental analysis. This is done due to the need to use a squared lipid patch with regularly positioned molecules of 4 Vpu to build the larger patch.

In this paper the dynamics of oligomerization of the 16mer is segregated into contributions of the transmembrane and the cytoplasmic domain in a quantitative way to parallel the experimental data set in respect of growth rate and maximum oligomerization ratio. In an earlier computational study structural features of the assembly of two Vpu proteins either as Vpu-WT and Vpu-DD are reported^31^. The sequence of occurrence of individual oligomers of Vpu-WT and Vpu-DD during the simulation of lipid patches with up to 16mers and 36mers is explored on a qualitative level.

### The sequence of protein assembly

The computational models were built alongside a biological pathway^6^. It is assumed that there is an equilibration of the monomeric unit of the membrane protein first, due to the distance between ribosomes (e.g., 500 Å apart from each other)^3^. The structure of the protein obtained in this state can be considered to be a ‘molten globule’ or ‘compact intermediate’, an intermediate state before the formation of a fully functional channel^3^. In a subsequent step larger assemblies are formed. A general feature is that the assembly of the host channels is in the minute to hour range^41^. Considerable time is dedicated to the folding of the subunits, a feature that is not explicitly considered in this study in as much CGMD simulation restrains the structure in its internal dynamics.

**Figure 7:**
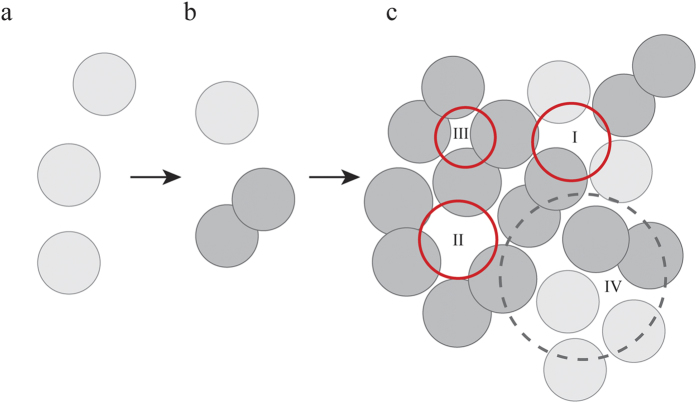
A schematic of the early event of putative assembly dynamics of 19 Vpu protein. Some of the individual monomers (**a**) shown as spheres in light grey assemble into dimers (dark grey spheres) in the first place (**b**). Within a larger assemble or patch (**c**), dimers and additional monomers are able to adopt specific conformations. These conformations can either be a channel-like state as marked by the red circles or not channel-like (marked by the dashed grey circle). Conformational changes of the proteins will allow transitions between the two states. Channel-like regions can exist as pentameric (I) or eventually hexameric (II) assembled Vpu proteins. A tetrameric assembly (III) of most likely two dimers could serve as precursor state of the formation of channel-like regions.

The experimental part of this study verifies a “dimer” first step of oligomerization of Vpu as simulated in an earlier study^31^. This formation of a dimer is driven by the association of the TMDs as indicated from computer simulations. In the dimer the two phosphorylation sites are the furthest apart due to electrostatic charge repulsion. During assembly into larger units the exposed negative charges of the phosphate groups have to be taken care of. Whilst the cytoplasmic domain directs oligomerization, the TMDs are responsible for holding the oligomer together. Based on this study, how Vpu is assembled and how it eventually reaches a pore-like structure is shown in the schema in Fig. 7. Some of the individual monomers assemble into dimers via association of TMDs (Fig. 7a,b). Within a larger assembly or patch (Fig. 7c), dimers and additional monomers are able to adopt conformations, which can either be channel-like (as marked by the red circles) or not channel-like (as marked by the dashed grey circles). Conformational changes of the proteins will allow e.g., to the transformation of Vpu proteins from the not channel-like region into the channel-like regions. It is always possible that the assemblies can be made out of the dimers or a mixture of both dimers and monomers. The generation of protein patches for more than 16–20 proteins may be restricted due to thermodynamic considerations taking into account protein binding affinities and protein dynamics due to the membrane environment.

The phosphorylation sites are necessary for the role of Vpu in initiating the ubiquinone-dependent downregulation of the proteins to which it attaches. According to this study, those sites seem to have another role in the regulation of the assembly of Vpu itself. Whether the interaction of Vpu with host factors occurs with Vpu as a monomeric or dimeric unit still needs to be investigated. It is also possible that Vpu interacts with host proteins in its patch-like assembly.

Dimerization is generally an essential first step in the oligomerization of membrane proteins. Specific sites within the protein, such as the two phosphorylation sites in the cytoplasmic domain of Vpu, play a modulating role during the initial step of assembly whilst the TMD defines the stability of the oligomer.

In the special case of Vpu, the phosphorylated serines have an additional function. Besides functioning in the initiation of the downregulation of an attached host protein it also regulates the oligomeric state of Vpu.

## Material and Methods

### Plasmids, cells and transfection

Human codon optimized Vpu genes derived from HIV-1 strain NL4-3 (P05923: MQPIQIAIAA^10^ LVVAIIIAIV^20^ VWSIVIIEYR^30^ KILRQRKIDR^40^ LIDRLIERAE^50^ DSGNESEGEI^60^ SALVEMGVEM^70^ GHHAPWDIDD^80^ L) were synthesized by multiple overlapping polymerase chain reaction (PCR) and cloned into the expression vector pTT-strep-his8 harboring a thrombin cleavage site for removing the tags. The cytoplasm domain mutants of single mutated Vpu, Vpu-S52D and -S56D, as well as double mutated Vpu, Vpu-S52/56D and Vpu-S52/56N were generated by quick-change site-directed mutagenesis and overlapping PCR respectively, by standard methods using the Phusion-II polymerase (New England BioLabs). For the single mutants, the second serine site is still available for phosphorylation during protein expression. Vpu with the mutations was also expressed using the vector pTT-strep-his8. All constructs were verified by sequencing analysis.

### Protein expression

Human embryonic kidney (HEK) 293 cells were maintained under standard humidified conditions (37 °C and 5% CO_2_). The cells, medium and serum were purchased from Invitrogen. Plasmid transfection into suspension cells was performed with the Transfection System (Invitrogen) according to a previously reported protocol^42^.

### Protein purification

Starting from a 1-liter culture, Vpu expressing HEK 293 cells were lysed by suspending in buffer (0.05 M Tris, pH8, 0.15 M NaCl, 20% glycerol, 1 mM PMSF (phenylmethanesulfonylfluoride) and 1 ng/ml DNAase). The fully suspended cells were disrupted twice on ice by Microfluidizer (M-110 L). Cell lysate was centrifuged in a JA25.5 rotor (Beckman Coulter) at 12,000 g for 35 min at 4 °C to remove unbroken cells and debris. The supernatant was then centrifuged in a Ti 45 rotor (Beckman Coulter) at 40,000 rpm for 1 h at 4 °C to separate the membrane pellet.

Vpu membrane pellets were homogenized with 0.5% (wt/vol) lauryldimethylamine-oxide (LDAO) detergents and incubated overnight at 4 °C. Detergent-solubilized membrane proteins were separated from insoluble material by centrifugation for 40 min at 12,000 g at 4 °C in JLA10.5 (Beckman Coulter). The supernatant was subjected into Strep-Tactin resin column by gravity. The resin was initially washed with high-salt buffer (0.05 M Tris, pH 8, 0.5 M NaCl, 1 mM ethylene diamine tetra acetate (EDTA), 0.05% (wt/vol) LDAO), then a low-salt buffer (0.05 M Tris, pH 8, 0.15 M NaCl, 1 mM EDTA, 0.05% (wt/vol) LDAO) and eluted with low-salt buffer containing 2.5 mM desthiobiotin (Sigma-Aldrich). The eluted protein was concentrated using an Amicon Ultra Centrifugal Filter (Millipore). The encoded protein contains a thrombin cleavage site (LVPRGS motif) cleavage site separating Strep-His8 tag from the C terminus of Vpu. Thus, a ratio of Thrombin (Sigma-Aldrich) was added to purified Vpu proteins at room temperature overnight. The mixture was concentrated using an Amicon Ultra Centrifugal Filter (Millipore).

### Protein analysis

Protein concentration in the samples was quantified by Nanotrop (Thermo). Protein purity was analyzed by SDS-PAGE on 16% acrylamide gels, stained by Rapid Stain. Size-exclusion chromatography was performed on a FPLC system (AKTA, GE) using a Superdex 200 10/30 size exclusion column pre-equilibrated in the buffer (0.05 M Tris, pH 8.0, 0.15 M NaCl and 0.05% (wt/vol) LDAO). The flow rate was set at 0.5 ml/min. Absorbance at 280 nm was monitored and recorded. The fraction from the major peak was collected in 0.5 ml fractions.

### SEC/MALS measurement

The mass determination of purified Vpu protein and LDAO detergent in Vpu-LDAO micelle was measured by a combination of size exclusion chromatography (SEC) coupled with three detectors: Multi-Angle Light Scattering (MALS), Refractive Index Detection (RI) and UV_280nm_ absorbance (Wyatt Technology). The whole system was pre-equilibrated with 50 mM Tris (pH 8.0), 150 mM NaCl and 0.05% LDAO buffer. Vpu-LDAO micelles were injected into a Superdex 200 10/30 column at 0.3 ml/min in buffer and then passed through the multi-angle light scattering detector and refractive index detector continuously. The data was analyzed by ASTRA software (Wyatt Technology).

### Construction of full-length Vpu model

An ideal helical structure of the first 52 amino acids of Vpu (Vpu_1–52_, HV1S1, P19554) was generated using the MOE software suit (www.chemcomp.com). The sequence used for Vpu_1–52_ was:


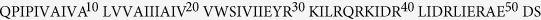


The helix was bent around residues Glu-28 to Ile-32 so that the helical stretch from residues Leu-33 to Ser-52 aligned with the membrane surface as described earlier^30^. Asp-39 was pointing towards the bilayer surface and Arg-48 was pointing into the aqueous phase, according to experimental findings^43^. In this configuration, the ϕ/ψ values of the amino acids in the bend are as follows: Glu-28: ϕ/ψ = −70.4°/0.2°, Tyr-29: ϕ/ψ = −65.2°/−42.5°, Arg-30: ϕ/ψ = −84.7°/−13.8°, Lys-31: ϕ/ψ = −86.2°/10.0°, Ile-32: ϕ/ψ = −57.6°/−18.0°. Two of these structures (each 530 atoms including united atoms), were embedded in a hydrated lipid bilayer so that they were inverted to each other and simulated for 100 ns. The last frame of the 100 ns MD simulation of Vpu_1–52_ (see red RMSD and RMSF curves in Supplementary Fig. S2) was chosen to generate full-length Vpu_1–81_.

The first structure out of the 20 structures of the models deposited in the PDB data bank (PDB ID: 2K7Y, HV1H2, P05919; residues 36 to 81)^16^, GSIDR_40_ LIDRITERAE^50^ DSGNESEGDQ^60^ EELSALVERG^70^ HLAPWDVDDL^80^, was chosen to be merged with Vpu_1–52_ from the MD simulations as follows. Residues Ile-39 to Arg-45, which adopt a helical motif, were merged with the helical motif of residues Ile-38 to Arg-44 of Vpu_1–52_ on the level of the Cα atoms to generate full-length Vpu, Vpu_1-80_ henceforth referred to as *Vpu-WT*.


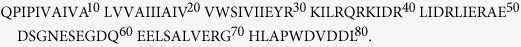


Two of these structures (790 atoms including united atoms) were embedded into a fully hydrated lipid bilayer as mentioned above. The last frame of the 100 ns MD simulation of Vpu_1-80_ (see red curves for root mean square deviation (RMSD) and root mean square fluctuation (RMSF) in Supplementary Fig. S3) was chosen to generate a coarse grained (CG) model *Vpu-WT*^31^.

A computational model of mutant *Vpu-DD*, was generated at the full-length structure prior to start the CGMD simulations by replacing Ser-52/56.

### Classical MD simulations

MD simulations on the systems reported in the present study were carried out with GROMACS 4.5.5 using Gromos96 (ffG45a3) force field with an integration step size of 2 fs. The temperature of the protein, lipid, and the water molecules were separately coupled to a Berendsen thermostat at 310 K with a coupling time of 0.1 ps. A semi isotropic pressure coupling was applied with a coupling time of 1.0 ps and a compressibility of 4.5e^−5^ bar^−1^. Long-range electrostatics were calculated using the particle-mesh Ewald (PME) algorithm with grid dimensions of 0.12 nm and interpolation order 4. Lennard-Jones and short-range Coulomb interactions were cut off at 1.4 and 1 nm, respectively.

Vpu_1–52_ and Vpu_1–80_ proteins were put on either side of the lipid bilayer consisting of 228/228 lipids (11856/11856 atoms) and hydrated with 11671/11473 water molecules (35013/34419 atoms) (Supplementary Figs S2 and S3). Lipids which overlapped with the peptide were removed. The system was then minimized (5000 steps of steepest descent and 5000 steps of conjugate gradient) and equilibrated for a total of 8.65 ns. Equilibration was achieved by gradually increasing the temperature from 100 K to 200 K and then to 310 K, whilst keeping the peptide fully restrained (k = 1000 kJ mol^−1^ nm^−2^). The first two simulations (at 100 K and 200 K) were run for 200 ps, the last simulation (at 310 K) was run for 8.5 ns. It was verified that the space between helix 2 and lipid membrane did not contain any water molecules, since the hydrophobic residues were pointing toward to the lipids. Holding the system at 310 K, the restraints, imposed by a force constant k on the peptide, were released in two steps (k = 500 kJ mol^−1^ nm^−2^, k = 250 kJ mol^−1^ nm^−2^), running each of the steps for 500 ps. The unconstrained systems were submitted to production runs of 100 ns.

The last frame of the 100 ns MD simulation of Vpu_1–80_ was used to replace the serines at site 52 and 56 into aspartic acid (Vpu_1–80_-DD).

### Coarse-grained MD simulations

Coarse-grained molecular dynamics (CGMD) simulations using the Gromacs software were performed using the MARTINI force field v2.0 for water and v2.1 for protein^44^,^45^. The Martini script was used to convert Vpu_1–80_ and Vpu_1–80_-DD into coarse-grained structural models. A default elastic network was used^46^. The integration time step was Δt = 30 fs and periodic boundary conditions were applied. The non-bonded interaction had a cut off distance of 1.2 nm. The temperature of the protein, lipid, and the water molecules were separately coupled to a Berendsen thermostat at 310 K with a coupling time of 1.0 ps. A semi-isotropic pressure coupling was applied with a coupling time of 12.0 ps and a compressibility 3e-5 bar^–1^. For the lipid bilayer, a pre-equilibrated 2048 lipid POPC membrane hydrated by 33912 water molecules was used as a starting point. Sixteen full-length Vpu and Vpu-DD mutants were embedded in a POPC membrane (Supplementary Fig. S4) with a protein: lipid ratio of 1:9^31^. Na-ions were added to neutralize the system. The systems consisted of 57064 and 57058 beats for the *Vpu-WT* and *Vpu-DD* system, respectively. The simulations with mixtures of *Vpu-WT* and *Vpu-DD* were generated by replacing 4 Vpu proteins in a row by the other type of protein. The simulation systems were then neutralized with the respective number of Na-ions. All the system were energy minimized (500 steps of steepest decent) and equilibration with protein restrain (k = 500 kJ mol^−1^ nm^−2^) for a total of 2.7 ns. The unrestrained systems were submitted to production runs of 10 μs.

### Oligomerization analysis

The oligomerization rate of the computational data was calculated with the concentration of trimer or higher oligomers divided by the concentration of total oligomer, in order to quantify the oligomerization level the maximum value was 1 and the minimum was 0. The respective curves were fitted with a logarithmic growth function.


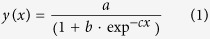


with a = maximum oligomerization ratio, b = initial value at time t = 0, and c = growth rate (time^−1^). Non-linear regression was performed by using non-linear curve fitting of OriginLab 9.0. The initial values were set to a = 1, b = 1, and c = 0.1. Iteration was conducted until the difference between reduced χ^2^ values of two successive iterations was less than a specified tolerance value, here 10^−9^ by default. Fitting the experimental Vpu-WT data two logistic growth functions were combined additively.

Structures are considered as oligomers when the distance between 10 pairs of CG-atoms of different Vpu structures was below 5 Å and observed continuously for more than 10 times steps between the proteins.

## Additional Information

**How to cite this article**: Chen, C.-P. *et al.* Membrane protein assembly: two cytoplasmic phosphorylated serine sites of Vpu from HIV-1 affect oligomerization. *Sci. Rep.* **6**, 28866; doi: 10.1038/srep28866 (2016).

## Supplementary Material

Supplementary Information

## Figures and Tables

**Table 1:** Relevant data from three different SEC/MALS experiments.

	Experiment 1		Experiment 2		Experiment 3		Avg σ (n−1)	
	Peak 1	Peak 2	Peak 1	Peak 2	Peak 1	Peak 2	Peak 1	Peak 2
MW [kDa]	9.2	9.2	9.2	9.2	9.2	9.2	–	–
Polydispersity (Vpu)	1.001 (1%)	1.003 (3%)	1.002 (2%)	1.005 (5%)	1.002 (2%)	1.009 (9%)	–	–
Conjugated MW [kDa]	309.4	44.17	316.5	45.59	282.24	48.48	302.7 ± 18.1	46.1 ± 2.2
Detergent MW [kDa]	122.6	28.04	132.8	27.58	128.9	28.64	128.1 ± 5.1	28.1 ± 0.5
Vpu MW [kDa]	186.9	16.13	183.7	18.01	153.5	19.85	174.7 ± 18.4	18.0 ± 1.9
Oligomeric state	20.31	1.75	20.05	1.96	16.68	2.15	19.0 ± 2.0	2.0 ± 0.2

MW = molecular weight in kDa. Avg = averaged values from the three experiments.

**Table 2:** Fitting parameters using a logistic growth function (
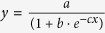
) where y is the rate over time, here x.

Experimental	a	b	c (growth rate) [day^−1^]
Vpu-NN	0.78	1.86	1.05
Vpu-WT	0.29	93.56	2.35
	0.39	18.49	0.31
Vpu-DD	0.22	122.74	0.56
**Computational**	**a**	**b**	**c (growth rate) [μs^−1^]**
*Vpu-WT*	0.98	271.28	0.009
*Vpu-DD*	0.58	85.10	0.004
*Vpu-WT*			
cyto	027	395.50	0.014
TM	0.68	95.56	0.003
*Vpu-DD*			
cyto	0.07	1479.60	0.033
TM	0.50	102.34	0.004

The parameters are a = maximum oligomerization ratio, b = initial value at time t = 0, and c = growth rate (time^−1^). Two terms of the logistic growth function are combined additively to fit the experimental data of Vpu-WT.
