# Supplementary material for: Membrane protein assembly: two cytoplasmic phosphorylated serine sites of Vpu from HIV-1 affect oligomerization
Source: Sci Rep. 2016 Jun 29;6:28866. doi: 10.1038/srep28866 (PMC4926278; doi:10.1038/srep28866)
Supplement: Supplementary Information [file srep28866-s1.doc]

**Supplemental Material**

**Membrane protein assembly: two cytoplasmic phosphorylated serine sites of Vpu from HIV-1 affect oligomerization.**

Chin-Pei Chen^1^, Meng-Han Lin^1^, Ya-Ting Chan^2^, Li-Chyong Chen^3^, Che Ma^2^*, and Wolfgang B. Fischer^1^*

^1^Institute of Biophotonics, School of Biomedical Science and Engineering and Biophotonics & Molecular Imaging Center (BMIRC), National Yang-Ming University, Taipei 112, Tawan

^2^Genomics Research Center, Academia Sinica, Taipei 115, Taiwan

^3^Center for Condensed Matter Sciences, National Taiwan University, Taipei 106, Taiwan

*Corresponding authors: [wfischer@ym.edu.tw](mailto:wfischer@ym.edu.tw), [cma@gate.sinica.edu.tw](mailto:cma@gate.sinica.edu.tw)

**Keywords:** Vpu of HIV-1, oligomerization, dynamics, phosphorylation, protein assembly, coarse grained MD simulations

**Supplementary Table S1:** Vpu in its various forms and the proposed oligomeric states obtained by using the respective experimental techniques. SPPS = solid phase peptide sysnthesis; TFE = tetrafluoroethanol; NMR = nuclear magnetic resonance; AUC = analytical ultracentrifugation; *E. coli* = *Escherichia coli*; DHPC = 1,2-diheptanoyl-*sn*-glycero-3-phosphocholine;

|  | Oligomeric state | Generation of protein | Detergent | Technique | references |
| --- | --- | --- | --- | --- | --- |
| Vpu_1-40_ | 4 to 6 | SPPS | Dissolved in TFE | NMR, AUC, photo-induced crosslinking |  |
| Vpu_2-32_ | 4 or 5 | *E. coli* | DHPC | Gel electrophorese |  |
| ^35^S labeled full length Vpu | 5 | Coupled transcription/translation system | - | Gel permeation chromatography |  |

**Supplementary Table S2:** Fitting parameters using a double logistic growth function ($y=\frac{a}{\left(1+b\cdot e^{-cx}\right)}+\frac{d}{\left(1+e\cdot e^{-fx}\right)}$ ) where y is the rate over time, here x. The parameters are a/d = maximum oligomerization level (%), b/e = initial value at time t = 0, and c/f = growth rate (time^-1^). In the mixtures, *Vpu-WT* is indicated as ‘*WT*’ while *Vpu-DD* is indicated as ‘*DD*’. Section TMD represents the values by fitting the oligomerization ratio of the TMD only.

| parameters | a | b | c |
| --- | --- | --- | --- |
| mixtures | d | e | f |
| 12 *WT* / 4 *DD* | 0.46 | 26302 | 0.01702 |
|  | 0.54 | 91.5 | 0.01754 |
| 8 *WT* / 8 *DD* | 0.27 | 133317 | 0.0068 |
|  | 0.73 | 41.9 | 0.0029 |
| 4 *WT* / 12 *DD* | 0.47 | 12487.9 | 0.0182 |
|  | 0.52 | 6.0 | 0.0099 |

TMD

| parameters | a | b | c |
| --- | --- | --- | --- |
| mixtures | d | e | f |
| 12 *WT* / 4 *DD* | 0.48 | 25.4 | 0.0036 |
|  | 0.19 | 991483 | 0.0045 |
| 8 *WT* / 8 *DD* | 0.61 | 27.2 | 0.00208 |
|  | 0.38 | 772584 | 0.0047 |
| 4 *WT* / 12 *DD* | 0.23 | 466.1 | 0.0097 |
|  | 0.20 | 357437 | 0.01856 |


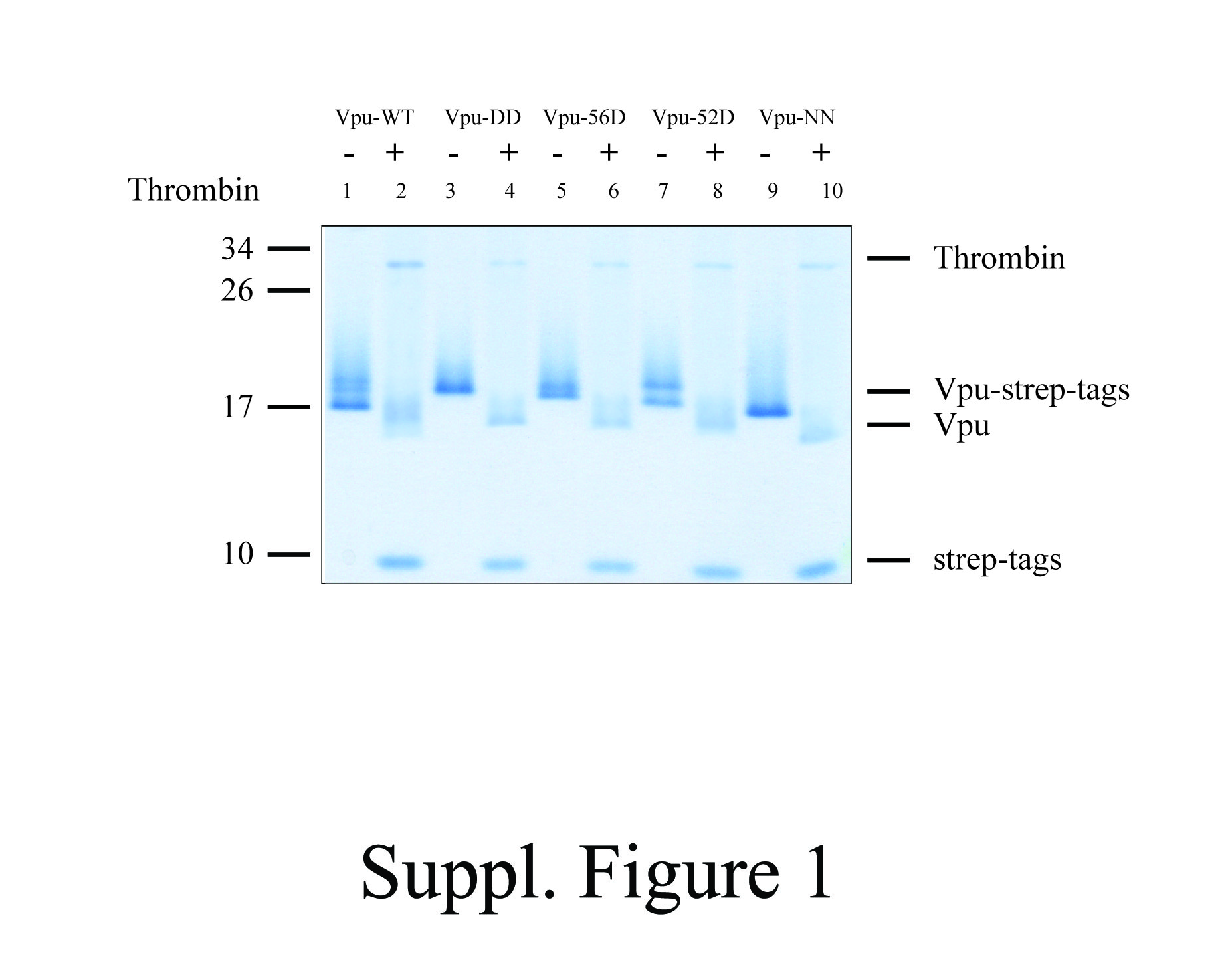


**Supplementary Fig. S1:** Image enhanced picture of Figure 1A to visualize band patterns of the throbin treated lanes 2, 4, 6, 8, 10. Two oligomeric states of wild-type and different mutants of full-length Vpu (pNL4.3) from HIV-1 SDS-PAGE analysis of purified Vpu-WT, Vpu-DD, Vpu-56D, Vpu-52D, Vpu-NN protein elution from left to right (A). All fusion proteins were treated with thrombin enzyme to remove the fusion strep-tags. The “+” and “-” represent Vpu fusion proteins with or without the addition of thrombin enzymes. Molecular mass is given in kDa.


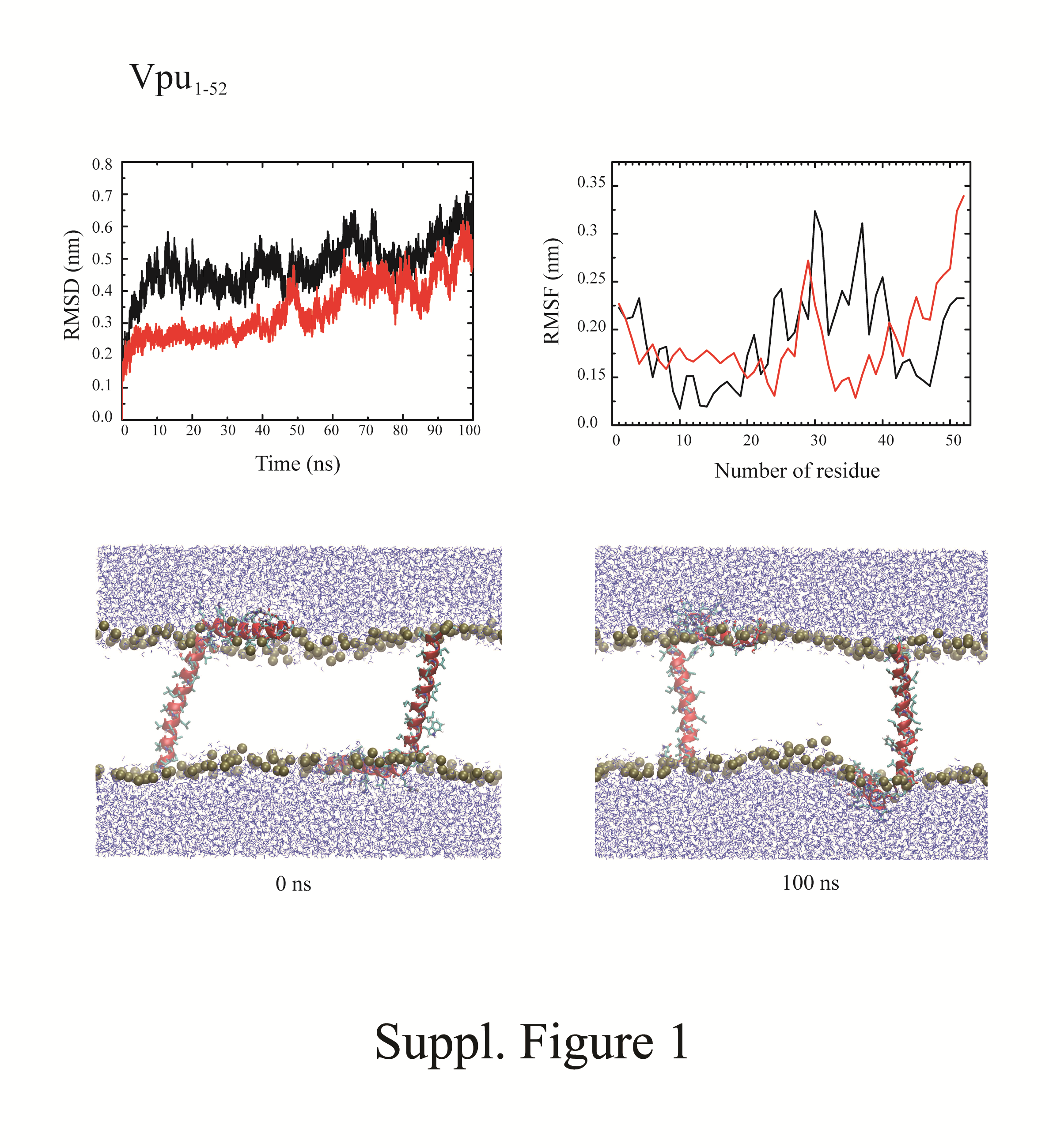


a

b

**Supplementary Fig. S2:** (a) Graphical representation of the time-dependent root mean square deviation (RMSD), left, and root mean square fluctuation (RMSF) values, right, of the bent Vpu_1-52_. (b) Structures of two Vpu_1-52_ inversely embedded into a lipid bilayer at the start (0 ns, left) and at the end of a 100 ns MD simulation (right). Backbone atoms are shown in red ribbon representation; the side chains are in grey stick modus. The boundaries of the lipid membrane are indicated by the phosphorous atoms of the lipid head groups shown in metallic van-der-Waals-spheres. The remaining atoms of the lipid molecule are omitted for clarity. Water molecules are shown as blue lines.


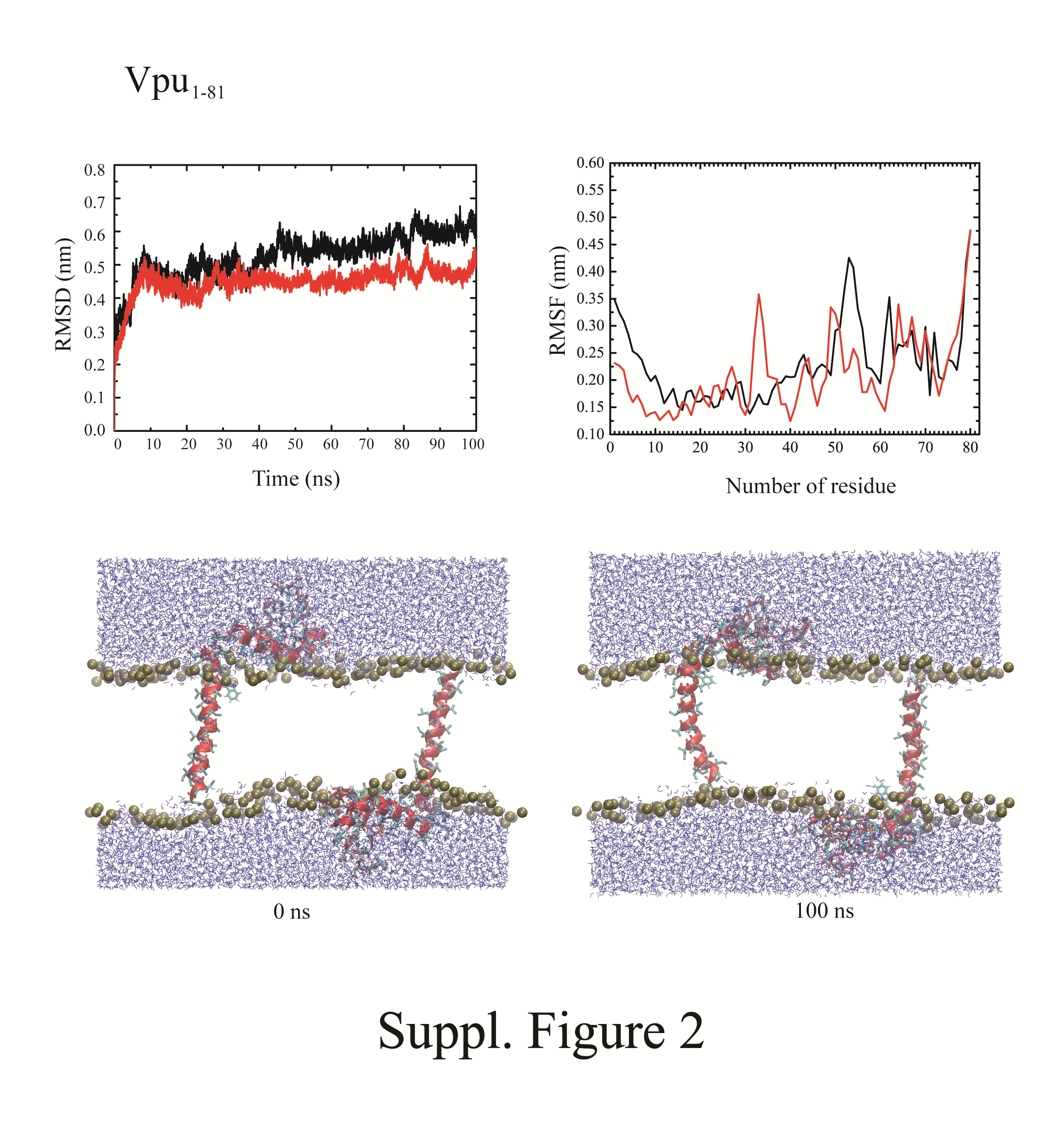


a

b

**Supplementary Fig. S3:** (a) Graphical representation of the time-dependent root mean square deviation (RMSD), left, and root mean square fluctuation (RMSF) values, right, of full-length Vpu, Vpu_1-80_. (b) Structures of two Vpu proteins inversely embedded into a lipid bilayer at the start (0 ns, left) and at the end of a 100 ns MD simulation (right). Backbone atoms are shown in red ribbon representation, the side chains in grey stick modus. The boundaries of the lipid membrane are indicated by the phosphorous atoms of the lipid head groups shown in metallic van der Waals spheres. The remaining atoms of the lipid molecule are omitted for clarity. Water molecules are shown as blue lines.


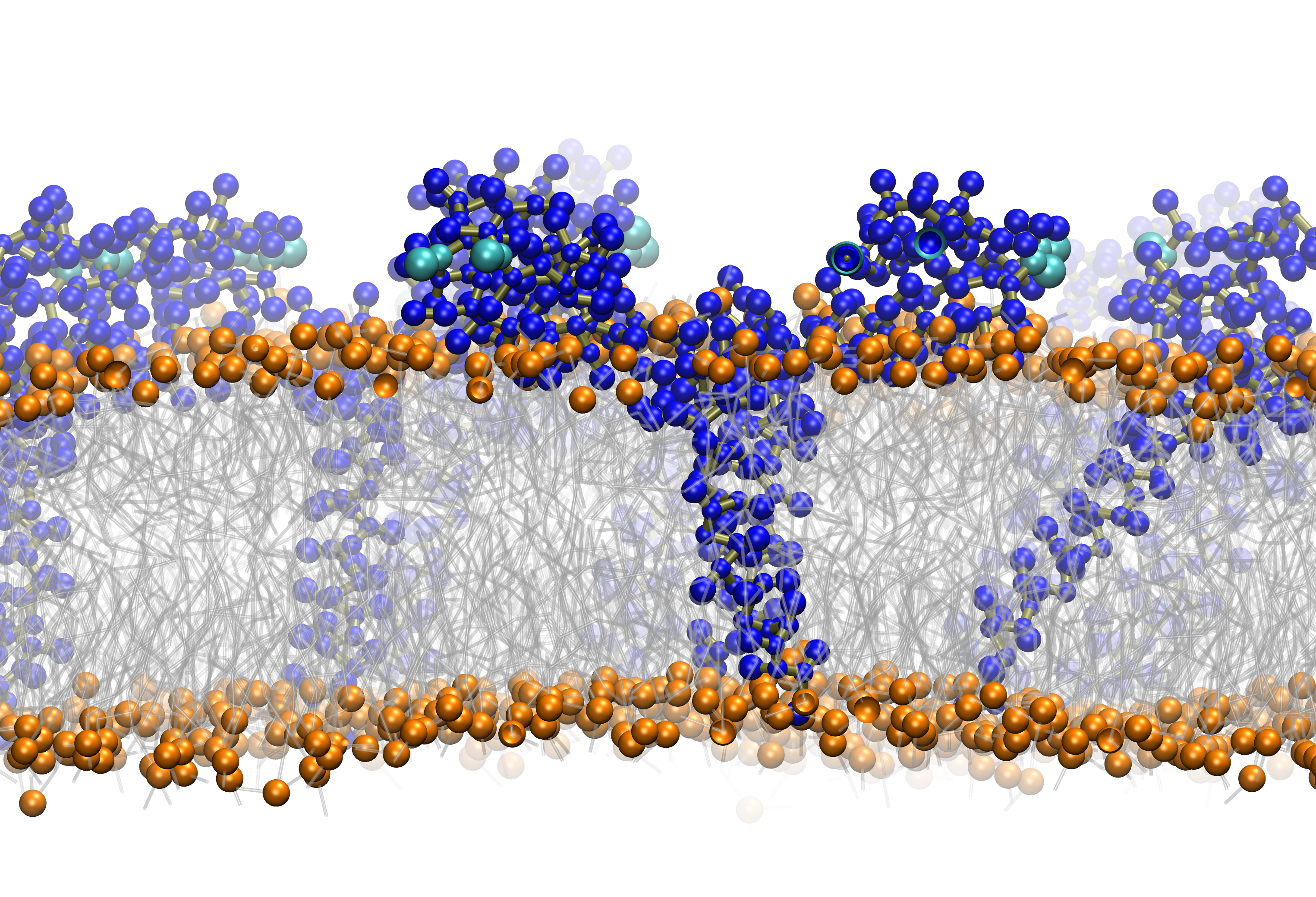


a

b


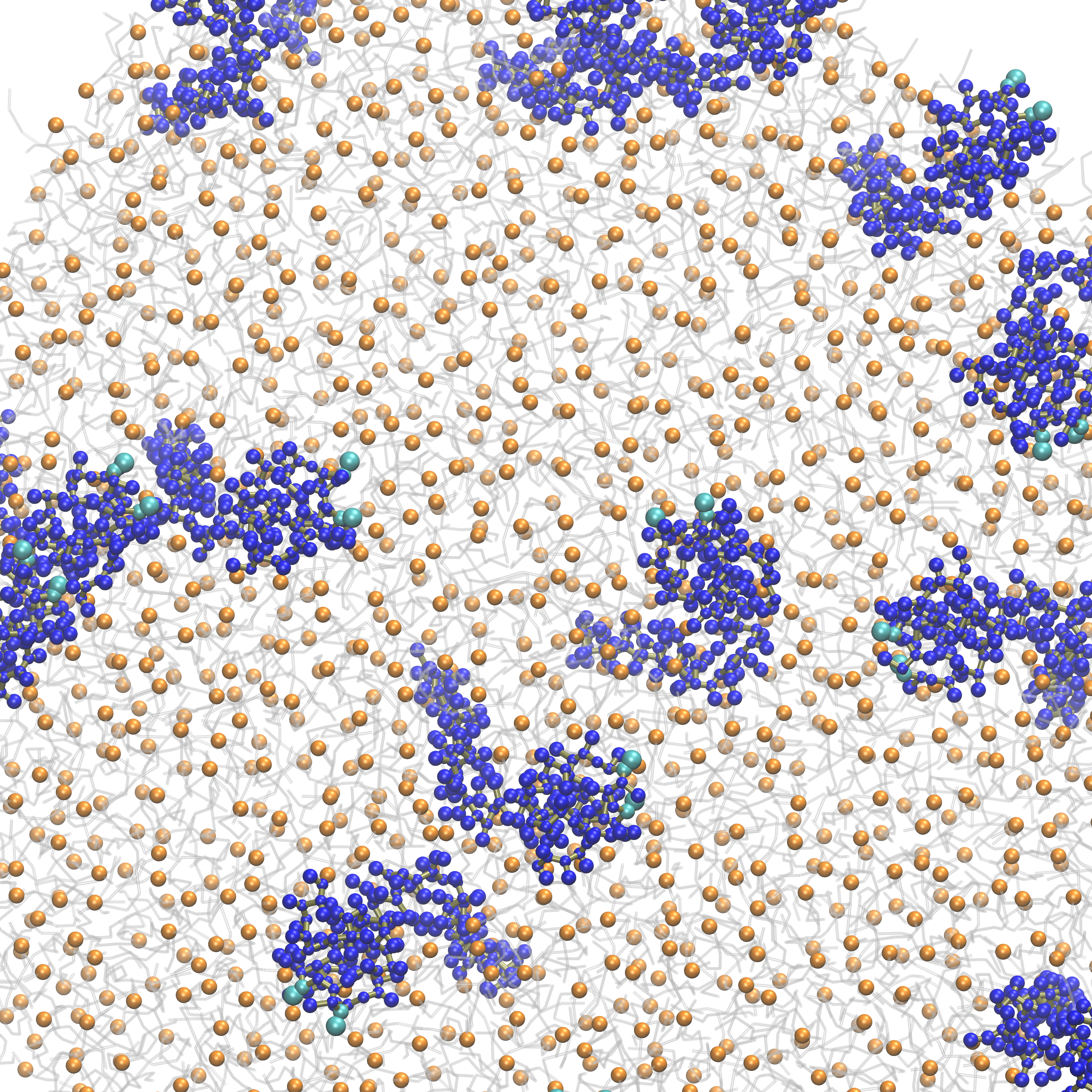


**Supplementary Fig. S4:** Snapshot taken at 300 ns of a Vpu-WT monomer existing temporarily during the CGMD simulation of 16 Vpu-WT (see Materials and Methods). The structures are shown in a side view (a) and a top view (towards the side of the cytoplasmic domain outside the membrane) (b) within the hydrated lipid bilayer to emphasis that the view in (a) represents that of a monomer. Serines, Ser-52 and -56 are shown in light blue spheres. The phosphate moieties of the lipid bilayer are shown in orange spheres to indicate the approximate dimension of the lipid bilayer. The hydrophobic tails are indicated by light grey lines. Spheres representing the water molecules are omitted for clarity.


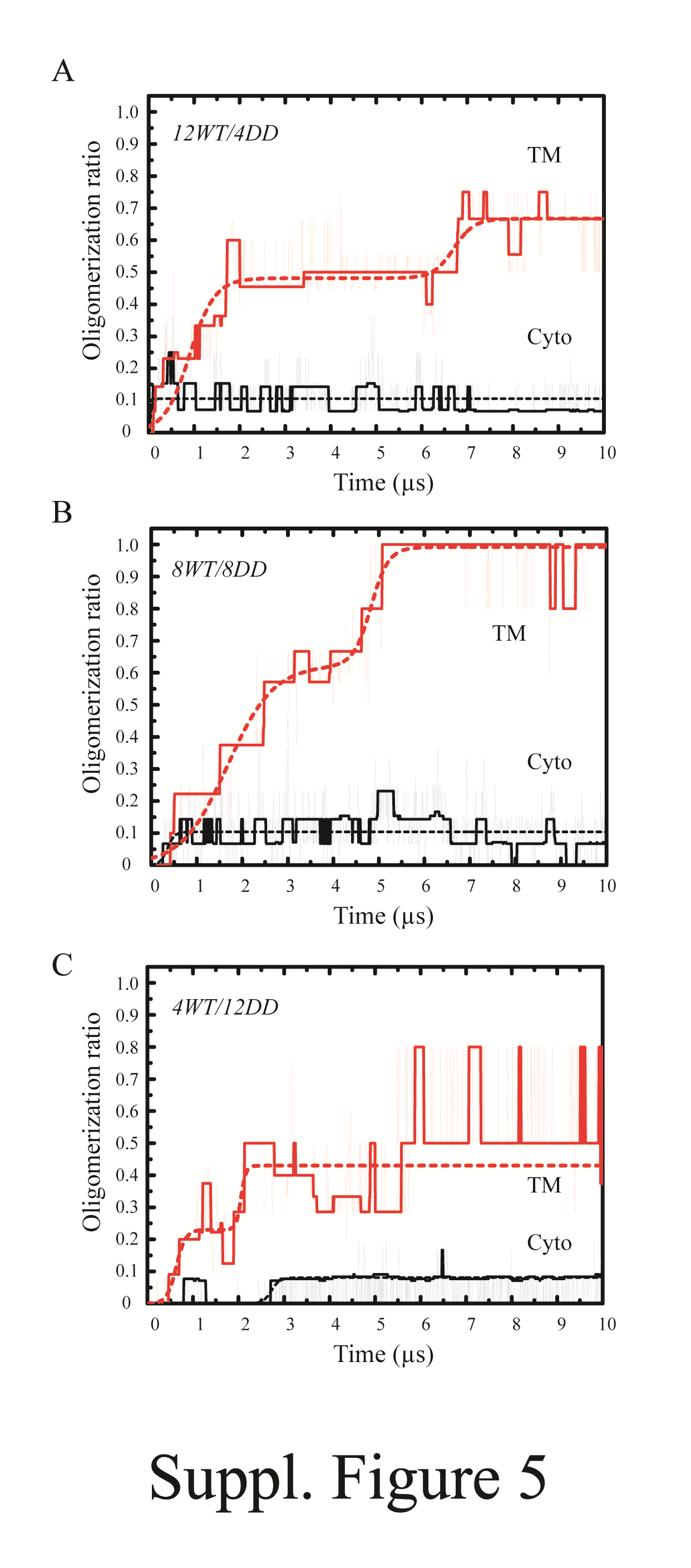


a

b

c

**Supplementary Fig. S5:** **Comparison of the oligomerization ratio from CGMD simulations** Time dependent representation of the oligomerization ratio of mixtures of *Vpu-WT* and *Vpu-DD*: 12 *Vpu-WT* and 4 *Vpu-DD* (12WT/4DD) (a), 8 *Vpu-WT* and 8 *Vpu-DD* (8WT/8DD) (b) as well as 4 *Vpu-WT* and 12 *Vpu-DD* (4WT/12DD) (c) in the simulation box. The total oligomerization ratio is separated into the ratio of the transmembrane domain (TM, red curves) and the cytoplasmic domains (cyto, black curves). Data are fitted with a double logarithmic curve (see Supplementary Table S2).

**Supporting** **References**

1. Sharpe, S., W. M. Yau, and R. Tycko. 2006. Structure and Dynamics of the HIV-1 Vpu Transmembrane Domain Revealed by Solid-State NMR with Magic-Angle Spinning. Biochemistry 45:918-933.

2. Park, S. H., A. A. Mrse, A. A. Nevzorov, M. F. Mesleh, M. Oblatt-Montal, M. Montal, and S. J. Opella. 2003. Three-dimensional structure of the channel-forming trans-membrane domain of virus protein "u" (Vpu) from HIV-1. J. Mol. Biol. 333:409-424.

3. Hussain, A., S. R. Das, C. Tanwar, and S. Jameel. 2007. Oligomerization of the human immunodeficiency virus type I (HIV-1) Vpu protein - a genetic, biochemical and biophysical analysis. Virol. J. 4:1-11.
